#  Depressão, ansiedade, estresse e apoio social: estudo transversal com cuidadores de crianças com deficiência visual no Rio de Janeiro, Brasil - *Views-QoL* Study 

**DOI:** 10.1590/0102-311XPT247622

**Published:** 2023-12-15

**Authors:** Martha Cristina Nunes Moreira, Ricardo Ewbanck Steffen, Andrea Araujo Zin, Marisa da Silva Santos, Ana Carolina Carioca da Costa, Daniel de Souza Campos, Letícia Baptista de Paula Barros, Maria Elisabeth Lopes Moreira, Corina Helena Figueira Mendes, Hannah Kuper, Márcia Pinto

**Affiliations:** 1 Instituto Nacional de Saúde da Mulher, da Criança e do Adolescente Fernandes Figueira, Fundação Oswaldo Cruz, Rio de Janeiro, Brasil.; 2 Instituto de Medicina Social, Universidade do Estado do Rio de Janeiro, Rio de Janeiro, Brasil.; 3 Instituto Nacional de Cardiologia, Rio de Janeiro, Brasil.; 4 Escola de Serviço Social, Universidade Federal do Rio de Janeiro, Rio de Janeiro, Brasil.; 5 London School of Hygiene & Tropical Medicine, London, U.K.

**Keywords:** Saúde Mental, Cuidadores, Transtornos da Visão, Apoio Social, Mental Health, Caregivers, Vision Disorders, Social Support, Salud Mental, Cuidadores, Trastornos de la Visión, Apoyo Social

## Abstract

O objetivo foi identificar os relatos de sintomas de depressão, ansiedade e estresse entre cuidadores de crianças sem deficiência visual, com baixa visão e com cegueira e sua relação com o grau de apoio social, emocional, material e afetivo. Estudo transversal e multicêntrico, realizado no Município do Rio de Janeiro, Brasil, entre 2019 e 2020. Aplicou-se um questionário para obter dados sociodemográficos e econômicos do cuidador. Foram utilizadas a *Escala de Apoio Social* (*The Medical Outcomes Study Social Support Scale -* MOS-SSS) e a *Escala de Depressão, Ansiedade e Estresse* (*The Depression, Anxiety, and Stress Scale -* DASS-21). Na comparação entre as escalas, foram utilizados testes para comparações múltiplas. Estimou-se a razão de prevalência de sintomas de depressão, ansiedade e estresse. Do total de cuidadores (N = 355), mais de 90% eram mulheres-mães e a maior proporção de cuidadores sem instrução ou Ensino Fundamental incompleto e com menor renda média mensal foi daqueles de crianças com deficiência visual. A maioria dos cuidadores de crianças com cegueira relatou sintomas de depressão, ansiedade e estresse (respectivamente, 66,7%, 73,3% e 80%), mesmo comportamento observado no grupo de cuidadores de crianças com baixa visão. Na avaliação da relação entre os resultados das escalas MOS-SSS e DASS-21, entre os cuidadores de crianças sem deficiência ou com menor comprometimento visual, observou-se maiores apoios e menores escores de relatos de depressão, ansiedade e estresse. Entre os cuidadores de crianças cegas, as maiores prevalências não dependeram dos apoios recebidos. Os resultados indicam a necessidade de uma política de cuidado com mecanismos de proteção à saúde mental dos cuidadores de crianças com deficiência visual.

## Abstract (en)

We aimed to identify the reports of symptoms of depression, anxiety, and stress among caregivers of children without visual impairment, with low vision, and with blindness and their relationship with the degree of social, emotional, material, and affective support. This cross-sectional and multicenter study was conducted in the municipality of Rio de Janeiro, Brazil, from 2019 to 2020. A questionnaire was applied to obtain caregivers’ sociodemographic and economic data. The *Medical Outcomes Study Social Support Scale* (MOS-SSS) and *The Depression, Anxiety, and Stress Scale* (DASS-21) were used. Tests were used for multiple comparisons of these scales. The prevalence ratio of symptoms of depression, anxiety, and stress was estimated. Of all caregivers (N = 355), more than 90% were women-mothers. Caregivers of children with visual impairment show the highest proportion of no schooling, incomplete elementary education, or lower average monthly income. Most caregivers of children with blindness reported symptoms of depression, anxiety, and stress (66.7%, 73.3%, and 80%, respectively) as did those of children with low vision. The evaluation of the relationship between MOS-SSS and DASS-21 results shows greater support and lower scores of reports of depression, anxiety, and stress for caregivers of children without disabilities or with less visual impairment. For caregivers of blind children, the highest prevalence of such reports was independent of the received support. Results indicate the need for a care policy with mechanisms to protect the mental health of caregivers of visually impaired children.

## Resumen

El objetivo fue identificar los relatos de síntomas de depresión, ansiedad y estrés entre cuidadores de niños sin discapacidad visual, con baja visión y con ceguera y su relación con el grado de apoyo social, emocional, material y afectivo. Estudio transversal y multicéntrico realizado en la ciudad de Río de Janeiro, Brasil, entre el 2019 y el 2020. Se aplicó un cuestionario para obtener datos sociodemográficos y económicos del cuidador. Se utilizaron la *Escala de Apoyo Social* (*The Medical Outcomes Study Social Support Scale* - MOS-SSS) y *Escala de Depresión, Ansiedad y Estrés* (*The Depression, Anxiety, and Stress Scale* - DASS-21). Al comparar las escalas, se utilizaron pruebas para comparaciones múltiples. Se estimó la razón de prevalencia de síntomas de depresión, ansiedad y estrés. Del total de cuidadores (N = 355), más del 90% eran mujeres madres y la mayor proporción de cuidadores sin escolaridad o con primaria incompleta y con menor ingreso mensual promedio fueron los de niños con discapacidad visual. La mayoría de los cuidadores de niños con ceguera reportó síntomas de depresión, ansiedad y estrés, respectivamente, 66,7%, 73,3% y 80%, mismo comportamiento observado en el grupo de cuidadores de niños con baja visión. Al evaluar la relación entre los resultados de las escalas MOS-SSS y DASS-21, entre los cuidadores de niños sin discapacidad o con menor compromiso visual, se observó mayor apoyo y menores puntajes de relatos de depresión, ansiedad y estrés. Entre los cuidadores de niños ciegos, la mayor prevalencia de tales relatos no dependió del apoyo recibido. Los resultados indican la necesidad de una Política de Cuidado con mecanismos para proteger la salud mental de los cuidadores de niños con discapacidad visual.

## Introdução

Estimativas globais indicam que no mínimo 2,2 bilhões de pessoas têm alguma condição associada à deficiência visual ^1^. A maior parcela dessas pessoas vive em países de baixa e média renda ^2^. Desse total, aproximadamente, metade dos casos seriam evitáveis se a organização dos serviços de saúde se estruturasse para que a oferta de tecnologias de prevenção, diagnóstico e tratamento atendesse de maneira eficiente e ágil a demanda por tais serviços ^3^. No Brasil, estima-se que, nos centros urbanos, as principais causas de deficiência visual sejam a retinopatia da prematuridade, as doenças infecciosas, anormalidades do nervo óptico, catarata e glaucoma ^4^^,^^5^.

A deficiência visual em crianças se expressa, conforme a gravidade, em atributos que afetam a qualidade de vida, como a cognição, a deambulação, a dor e a emoção ^6^. As causas de deficiência visual não ocorrem sozinhas e, frequentemente, vêm acompanhadas de outras condições crônicas ^7^^,^^8^. As crianças com deficiência visual apresentam menores índices de desempenho escolar e menor autoestima quando comparadas àquelas sem deficiência visual ^9^^,^^10^. Observa-se, também, problemas de linguagem e de desenvolvimento neuromotor relacionados ao atraso no aprendizado escolar, além de dificuldades no desempenho funcional nas áreas de autocuidado e mobilidade ^7^^,^^11^.

Entre os cuidadores de crianças com alguma deficiência, a prevalência de depressão e ansiedade mostrou-se mais elevada entre aqueles com dificuldades funcionais importantes ^12^^,^^13^. Essas questões relacionadas à saúde mental foram potencializadas quando esses cuidadores vivenciaram algum tipo de discriminação em relação às crianças ^12^^,^^13^^,^^14^. Ainda, há a sobrecarga do cuidador diante de uma rotina de exaustão física e mental, que leva ao estresse e interfere também no seu bem-estar ^14^.

A rotina de cuidados de uma criança com deficiência também pode afetar a saúde física do seu responsável, gerando cansaço, poucas horas de sono, dores no sistema musculoesquelético e ampliando as repercussões econômicas e sociais, como a incidência de gasto catastrófico, redução do número de horas trabalhadas, dificuldade de inserção no mercado de trabalho e impossibilidade de realizar atividades sociais ^15^^,^^16^. Esses fatores podem contribuir para aumentar o risco de os cuidadores de crianças com deficiência visual desenvolverem sintomas de depressão, ansiedade e estresse ^17^^,^^18^^,^^19^.

O apoio social é fundamental para a sustentação das relações de cuidado, com destaque para aquelas que envolvem pessoas com deficiência. A literatura mostra que a presença de laços de ajuda mútua e de apoio social atua como fator protetor e sua ausência ou esgarçamento/enfraquecimento podem incrementar o risco de adoecimentos de outras ordens ^20^^,^^21^.

Diante desse contexto de vulnerabilidades nas famílias de crianças com deficiência visual, este artigo investigou a ocorrência de sintomas de depressão, ansiedade e estresse e de possíveis apoios sociais, emocionais, afetivos e materiais em relação aos cuidadores dessas crianças em centros de referência em doenças oculares localizados no Município do Rio de Janeiro, Brasil.

## Métodos

Estudo transversal, multicêntrico e que faz parte da pesquisa *Triagem de Doenças Oculares em Recém-nascidos e Perda de Qualidade de Vida por Cegueira ou Baixa Visão em Crianças: Uma Análise sob as Perspectivas Econômica e Social* (Views-QoL Study; https://linktr.ee/ViewsQOL). Foi conduzido entre 2019 e 2020 em cinco centros no Município do Rio de Janeiro que recebem pacientes de diversas regiões do estado, dos quais três são vinculados ao Sistema Único de Saúde (SUS): um hospital federal e um hospital universitário estadual, ambos com serviços de referência em oftalmologia, e um hospital federal de referência em saúde da criança. Incluiu-se, também, uma instituição federal especializada no ensino de pessoas com deficiência visual e uma organização não governamental (ONG) que fornece apoio a famílias de baixa renda e atende prioritariamente crianças com diagnóstico de catarata infantil, por meio de uma rede de voluntários especializados na prestação de serviços de saúde.

### Recrutamento de casos e controles

A coleta de dados do estudo iniciou-se no segundo semestre de 2019 e foi suspensa em março de 2020, devido à pandemia de SARS-CoV-2. Em agosto do mesmo ano, a pesquisa foi retomada, mas, com o agravamento da pandemia no Brasil, foi finalizada em dezembro de 2020 ^22^.

O cuidador principal foi definido como caso se fosse a pessoa responsável pela rotina da criança, tanto no acompanhamento aos serviços de saúde e na instituição escolar, como nos cuidados diários. O recrutamento foi conduzido no departamento de oftalmologia dos hospitais com serviços de referência e teve o apoio de oftalmologistas presentes no momento da entrevista, especializados no diagnóstico e tratamento de crianças com deficiência visual. A seleção de casos, na ONG, foi realizada pelo profissional médico responsável pelo gerenciamento do caso após a confirmação diagnóstica. Na instituição de ensino, essa seleção foi realizada pelo contato pessoal com cuidadores das crianças com matrícula vigente nos anos deste estudo. Os cuidadores controle foram aqueles de criança sem deficiência, atendidos no hospital federal de referência em saúde da criança pelos profissionais de saúde responsáveis pela condução clínica.

Devido à pandemia de SARS-CoV-2, foi necessário incluir 121 participantes do estudo *Social and Economic Impacts of Zika Virus in Brazil* [Impactos Sociais e Econômicos do Vírus Zika no Brasil - estudo Seiz] ^23^, conduzido entre 2017 e 2019. Essa pesquisa foi aninhada ao estudo de coorte *Vertical Exposure to Zika Virus and It’s Consequences for Child Neurodevelopment: Cohort Study in Fiocruz/IFF* [*Exposição Vertical ao Vírus Zika e suas Consequências para o Desenvolvimento Neurológico de Crianças: Estudo de Coorte na Fiocruz/IFF*] (https://clinicaltrials.gov/, identificador: NCT03255369), realizado em um hospital de referência nacional em saúde da criança. Daquele total, 26 casos de cuidadores de crianças com deficiência visual e 95 cuidadores de crianças sem deficiência foram incorporados à amostra do Views-QoL Study.

Neste estudo, os mesmos instrumentos utilizados no estudo Seiz foram aplicados, e incluíram um questionário para a obtenção de dados sóciodemográficos, de escolaridade do cuidador, quantidade de pessoas que moravam na casa, idade e grau de parentesco com a criança, tipo de ocupação do cuidador (trabalhador formal, informal ou desempregado), status marital, e se recebia algum tipo de benefício oriundo de programas de transferência de renda, como o Benefício de Prestação Continuada (BPC) ou o Bolsa Família/Auxílio Emergencial. Estimou-se a renda do trabalho mensal das famílias e do cuidador pelo Critério Brasil ^24^, que propõe estratificar os domicílios por classes de renda. Os recursos recebidos oriundos de programas de transferência de renda foram excluídos, pois não representam a renda do trabalho. Esses benefícios se enquadram na categoria de apoio social, devendo ser objeto de análise separada.

A *Escala de Depressão, Ansiedade e Estresse* (*The Depression, Anxiety, and Stress Scale* - DASS-21) foi utilizada para investigar o relato de sintomas de depressão, ansiedade e estresse entre os cuidadores. A DASS-21 contém 21 itens, cujas respostas variam de 0 a 3 pontos. A presença de sintomas avaliada no questionário se refere à última semana e organiza-se em subescalas - depressão, ansiedade e estresse -, com sete itens para cada uma delas. Os sintomas são classificados por gravidade, de acordo com uma faixa de escores (normal, leve, moderado, grave ou muito grave). Para medir o apoio social recebido pelo cuidador, aplicou-se a escala *Escala de Apoio Social* (*The Medical Outcomes Study Social Support Scale* - MOS-SSS) que contempla as seguintes dimensões de apoio social: material, afetivo, interação social positiva, emocional e de informação. Ambos os instrumentos já foram validados no Brasil ^25^^,^^26^.

### Treinamento para a coleta de dados

Os entrevistadores que participaram dos estudos Seiz e Views-QoL Study foram treinados durante um dia mediante apresentação e discussão de cada item dos instrumentos. Um projeto piloto, com cinco entrevistas, foi realizado previamente ao início da coleta com o intuito de avaliar as dúvidas e problemas que poderiam surgir no decorrer de ambos os estudos.

### Análises estatísticas

A magnitude da associação entre as variáveis de exposição e o relato de sintomas de depressão, ansiedade e estresse foi quantificada em termos da razão de prevalência e seus respectivos intervalos de 95% de confiança (IC95%). Os dados coletados foram digitados e armazenados na plataforma FormSUS (http://siteformsus.datasus.gov.br/FORMSUS/index.php). Na análise descritiva, apresentou-se as frequências absolutas e porcentagens para caracterizar os dados categóricos, enquanto os dados numéricos foram descritos como média e desvio padrão ou mediana e intervalo interquartil. Os testes qui-quadrado, de Pearson, e exato de Fisher, foram utilizados para comparar as características sociodemográficas e econômicas dos casos e controles, a fim de identificar possíveis confundidores da associação entre os grupos de cuidadores e os desfechos de sintomas de saúde mental (depressão, ansiedade e estresse). Esses testes também foram usados para avaliar a relação entre as escalas MOS-SSS e DASS-21. Modelos de regressão múltipla de Poisson, com variância robusta, foram empregados para comparar as razões de prevalência de sintomas de depressão, ansiedade e estresse entre os três grupos (cuidadores de crianças com cegueira, baixa visão e sem deficiência visual), ajustados para os fatores de confundimento identificados nas análises bivariadas. Utilizou-se os softwares de análise de dados SPSS, versão 22 (https://www.ibm.com/), e R, versão 4.0.3 (http://www.r-project.org).

### Considerações éticas

O estudo seguiu os critérios da *Resolução nº 466/2012* do Conselho Nacional de Saúde e foi aprovado pelos Comitês de Ética em Pesquisa dos cinco centros participantes (CAAE nº 06814819.2.3005.5246). O estudo Seiz foi aprovado pelo Comitê de Ética em Pesquisa do Instituto Nacional de Saúde da Mulher, da Criança e do Adolescente Fernandes Figueira, Fundação Oswaldo Cruz (CAAE nº 60682516.2.1001.5269).

## Resultados

O total da amostra foi de 355 cuidadores, dos quais 45 (13%) de crianças com cegueira, 133 (37%) de crianças com baixa visão e 177 (50%) de cuidadores de crianças sem deficiência visual. Um total de 52,4% dos cuidadores declarou residir no Município de Rio de Janeiro. Nos grupos de cuidadores de crianças com cegueira e baixa visão, cerca de 56%, em cada grupo, residiam fora da cidade; em contrapartida, no grupo de famílias de crianças sem deficiência visual observou-se que mais de 60% viviam no Município do Rio de Janeiro.

Aproximadamente 38% dos cuidadores de crianças com cegueira relataram estar sem companheiro(a) no momento da entrevista, mais que o dobro dos cuidadores de crianças sem deficiência (18,6%) e 22,5% a mais que os cuidadores de crianças com baixa visão (30,8%). A maioria dos cuidadores eram mulheres-mães (91,5%) com idade entre 20 e 39 anos, e mais de 30% e 55% das crianças com cegueira e baixa visão, respectivamente, tinham até 5 anos. Entre os cuidadores de crianças com cegueira, 11% eram avós. O grupo responsável por crianças com cegueira tinha a maior proporção de cuidadores acima de 40 anos (29,5%). Aproximadamente metade dos cuidadores entrevistados completaram o Ensino Médio (50,7%), e os cuidadores de crianças com cegueira registraram o maior percentual sem instrução ou com a primeira etapa do Ensino Fundamental incompleta (13,3%), em comparação com aqueles de crianças com baixa visão (7,5%) e crianças sem deficiência visual (4%). No grupo de cuidadores de crianças com cegueira, a frequência de famílias com quatro moradores ou mais (31%) foi próxima do grupo de cuidadores de crianças com baixa visão e de crianças sem deficiência (32%) (Tabela 1).

**Tabela 1:** Características sociodemográficas e econômicas de cuidadores de crianças com cegueira, baixa visão e sem deficiência visual. Rio de Janeiro, Brasil.

Variável	Geral [n = 355] n (%)	Grupo [n (%)]			Valor de p
		Cegueira [n = 45]	Baixa visão [n = 133]	Sem deficiência visual [n = 177]	
Mora na cidade do Rio de Janeiro					0,005 *
Sim	186 (52,4)	20 (44,4)	58 (43,6)	108 (61,0)	
Não	169 (47,6)	25 (55,6)	75 (56,4)	69 (39,0)	
Situação conjugal					0,007 *
Com companheiro	264 (74,4)	28 (62,2)	92 (69,2)	144 (81,4)	
Sem companheiro	91 (25,6)	17 (37,8)	41 (30,8)	33 (18,6)	
Principal cuidador					0,132 **
Mãe	325 (91,5)	37 (82,2)	124 (93,2)	164 (92,7)	
Pai	7 (2,0)	1 (2,2)	3 (2,3)	3 (1,7)	
Avó	19 (5,4)	5 (11,1)	6 (4,5)	8 (4,5)	
Outro	4 (1,1)	2 (4,4)	-	2 (1,1)	
Idade do cuidador (anos)					0,042
< 20	10 (2,9)	2 (4,5)	4 (3,1)	4 (2,3)	
20-29	130 (37,4)	8 (18,2)	53 (40,5)	69 (39,9)	
30-39	139 (39,9)	21 (47,7)	45 (34,4)	73 (42,2)	
≥ 40	69 (19,8)	13 (29,5)	29 (22,1)	27 (15,6)	
Escolaridade					0,028 **
Sem instrução/Fundamental I incompleto	23 (6,5)	6 (13,3)	10 (7,5)	7 (4,0)	
Fundamental I completo/Fundamental II incompleto	52 (14,6)	5 (11,1)	19 (14,3)	28 (15,8)	
Fundamental II completo/Médio incompleto	63 (17,7)	9 (20,0)	29 (21,8)	25 (14,1)	
Médio completo/Superior incompleto	180 (50,7)	21 (46,7)	69 (51,9)	90 (50,8)	
Superior completo	37 (10,4)	4 (8,9)	6 (4,5)	27 (15,3)	
Número de moradores na residência					0,423 *
< 4	243 (68,5)	31 (68,9)	91 (68,4)	121 (68,4)	
4	69 (19,4)	10 (22,2)	21 (15,8)	38 (21,5)	
> 4	43 (12,1)	4 (8,9)	21 (15,8)	18 (10,2)	
Sexo da criança					0,296 *
Masculino	172 (48,5)	17 (37,8)	68 (51,1)	87 (49,2)	
Feminino	183 (51,5)	28 (62,2)	65 (48,9)	90 (50,8)	
Idade da criança (anos)					< 0,001 *
< 2	116 (32,7)	8 (17,8)	32 (24,1)	76 (42,9)	
2-5	121 (34,1)	6 (13,3)	42 (31,6)	73 (41,2)	
≥ 6	118 (33,2)	31 (68,9)	59 (44,4)	28 (15,8)	
Faixa de renda ***					0,001 *
A ou B	57 (16,1)	6 (13,3)	10 (7,5)	41 (23,2)	
C	206 (58,0)	24 (53,3)	80 (60,2)	102 (57,6)	
D ou E	92 (25,9)	15 (33,3)	43 (32,3)	34 (19,2)	
Ocupação (parou de trabalhar após nascimento da criança)	141 (39,7)	27 (60,0)	50 (37,6)	64 (36,2)	0,012 *
DASS-21					
Depressão					< 0,001 *
Não	172 (48,6)	15 (33,3)	52 (39,1)	105 (59,7)	
Sim	182 (51,4)	30 (66,7)	81 (60,9)	71 (40,3)	
Ansiedade					< 0,001 *
Não	200 (56,5)	12 (26,7)	55 (41,4)	133 (75,6)	
Sim	154 (43,5)	33 (73,3)	78 (58,6)	43 (24,4)	
Estresse					< 0,001 *
Não	191 (54,0)	9 (20,0)	51 (38,3)	131 (74,4)	
Sim	163 (46,0)	36 (80,0)	82 (61,7)	45 (25,6)	

DASS-21: *Escala de Depressão, Ansiedade e Estresse* (*The Depression, Anxiety, and Stress Scale*).

Fonte: elaboração própria.

* Teste qui-quadrado de Pearson;

** Teste exato de Fisher;

*** Faixas de renda média mensal, conforme o Critério Brasil: classe A = BRL 22.749,24; classe B = BRL 8.255,14; classe C = BRL 2.544,64; Classes D e E = BRL 862,41.

A distribuição por gênero foi similar entre os dois grupos, com 172 (48%) crianças do sexo masculino e 183 (52%) do sexo feminino. O mesmo resultado foi observado entre crianças com cegueira, invertendo-se entre as crianças com baixa visão, em que 51% eram do sexo masculino. O grupo responsável por crianças com cegueira tinha o maior número de crianças com idade ≥ 6 anos (62%) (Tabela 1).

A maior parte dos cuidadores era da classe de renda C (58%), com renda média mensal de BRL 2.544,64, e 16% eram das classes econômicas A ou B, com renda média de BRL 22.749,24 e BRL 8.255,14, respectivamente. O grupo de cuidadores de crianças sem deficiência visual foi o que apresentou o maior percentual de famílias que faziam parte das classes A ou B (23,2%), enquanto apenas 13,3% daqueles que cuidavam de crianças cegas faziam parte dessas duas classes. Mais de 32% dos cuidadores de crianças com cegueira ou baixa visão ganhavam cerca de BRL 862,00 ao mês (classes D e E), enquanto para os cuidadores de crianças sem deficiência esse percentual foi menor, de 19,2%. Após o nascimento da criança, aproximadamente 60% e 37% dos cuidadores de crianças com cegueira e baixa visão, respectivamente, pararam de trabalhar (Tabela 1).

A maioria dos cuidadores de crianças com cegueira relatou sintomas de depressão (66,7%), ansiedade (73,3%) e estresse (80%), resultado similar ao observado no grupo de cuidadores de crianças com baixa visão que apresentaram sintomas de depressão (60,9%), ansiedade (58,6%) e estresse (61,7%). No grupo de cuidadores de crianças sem deficiência visual ocorreu comportamento inverso, já que relataram a vivência dos sintomas citados em menor frequência (Tabela 1).

Os resultados dos modelos de regressão múltipla de Poisson com variância robusta sugeriram que os sintomas de depressão, ansiedade e estresse se tornaram mais frequentes à medida que o comprometimento da visão aumentava. A razão de prevalência ajustada de cuidadores de crianças com baixa visão e cegueira com sintomas de depressão foi, respectivamente, 40% e 50% maior que a observada no grupo de cuidadores de crianças sem deficiência. Também se observou que razões de prevalência foram superiores entre o grupo de cuidadores de crianças cegas e com baixa visão em relação ao relato dos sintomas de ansiedade e estresse (Tabela 2).

**Tabela 2:** Razões de prevalência de depressão, ansiedade e estresse ajustadas para os cuidadores de crianças sem deficiência visual, com baixa visão e com cegueira. Rio de Janeiro, Brasil.

Grupo	Depressão (≥ leve)			Ansiedade (≥ leve)			Estresse (≥ leve)		
	RP bruta (IC95%)	RP ajustada * (IC95%)	RP ajustada ** (IC95%)	RP bruta (IC95%)	RP ajustada * (IC95%)	RP ajustada ** (IC95%)	RP bruta (IC95%)	RP ajustada * (IC95%)	RP ajustada ** (IC95%)
Sem deficiência visual	1,0	1,0	1,0	1,0	1,0	1,0	1,0	1,0	1,0
Baixa visão	1,5 (1,2-1,9)	1,4 (1,1-1,7)	1,4 (1,1-1,8)	2,4 (1,8-3,2)	1,9 (1,4-2,6)	2,0 (1,5-2,7)	2,4 (1,8-3,2)	2,1 (1,5-2,8)	2,2 (1,6-2,9)
Cegueira	1,7 (1,3-2,2)	1,5 (1,1-2,0)	1,4 (1,0-1,9)	3,0 (2,2-4,1)	2,4 (1,6-3,4)	2,3 (1,6-3,3)	3,1 (2,3-4,2)	2,8 (2,0-3,8)	2,8 (2,0-3,8)

IC95%: intervalo de 95% de confiança; RP: razão de prevalência obtida através do modelo de Poisson com variância robusta.

Fonte: elaboração própria.

* Ajustada para cidade de moradia, situação conjugal, idade do cuidador, escolaridade do cuidador, idade da criança e classe socioeconômica;

** Ajustada para cidade de moradia, situação conjugal, idade do cuidador, escolaridade do cuidador, idade da criança, classe socioeconômica e apoio emocional, social, material e afetivo.

Na avaliação da relação entre os resultados da escala MOS-SSS e da escala DASS-21, nos grupos de cuidadores sem deficiência visual e com baixa visão, de uma forma geral, observou-se que quanto maior o grau de apoio emocional, social, material e afetivo, menor o percentual de cuidadores com sintomas de depressão, ansiedade e estresse. Entre os cuidadores de crianças com cegueira, observou-se as maiores prevalências de depressão, ansiedade e estresse, independentemente dos graus de apoio recebido (Tabela 3).

**Tabela 3:** Relato de sintomas de depressão, ansiedade e estresse e apoio emocional, social, material e afetivo entre cuidadores de crianças sem deficiência visual, com baixa visão e com cegueira. Rio de Janeiro, Brasil.

Apoio	Depressão [n (%)]			Ansiedade [n (%)]			Estresse [n(%)]		
	Sem deficiência visual	Baixa visão	Cegueira	Sem deficiência visual	Baixa visão	Cegueira	Sem deficiência visual	Baixa visão	Cegueira
Emocional									
Baixo	13 (59,1)	20 (90,9)	10 (83,3)	8 (36,4)	14 (63,6)	11 (91,7)	8 (36,4)	19 (86,4)	10 (83,3)
Médio	34 (45,3)	33 (67,3)	9 (56,3)	20 (26,7)	34 (69,4)	11 (68,8)	25 (33,3)	32 (65,3)	12 (75,0)
Alto	24 (30,4)	28 (45,2)	11 (64,7)	15 (19,0)	30 (48,4)	11 (64,7)	12 (15,2)	31 (50,0)	14 (82,4)
Valor de p	0,028 *	< 0,001 *	0,325 **	0,227 *	0,077 *	0,276 **	0,017	0,009	0,806 **
Social									
Baixo	15 (83,3)	16 (88,9)	10 (90,9)	8 (44,4)	15 (83,3)	10 (90,9)	8 (44,4)	17 (94,4)	10 (90,9)
Médio	28 (43,8)	34 (79,1)	8 (53,3)	14 (21,9)	27 (62,8)	11 (73,3)	17 (26,6)	29 (67,4)	11 (73,3)
Alto	28 (29,8)	31 (43,1)	12 (63,2)	21 (22,3)	36 (50,0)	12 (63,2)	20 (21,3)	36 (50)	15 (78,9)
Valor de p	< 0,001 *	< 0,001 *	0,136 **	0,143 **	0,029 *	0,248 **	0,120 **	0,001 *	0,575 **
Material									
Baixo	18 (54,5)	21 (80,8)	11 (68,8)	11 (33,3)	22 (84,6)	14 (87,5)	8 (24,2)	21 (80,8)	13 (81,3)
Médio	26 (40,6)	35 (62,5)	8 (72,7)	18 (28,1)	32 (57,1)	7 (63,6)	23 (35,9)	36 (64,3)	8 (72,7)
Alto	27 (34,2)	25 (49,0)	11 (61,1)	14 (17,7)	24 (47,1)	12 (66,7)	14 (17,7)	25 (49,0)	15 (83,3)
Valor de p	0,134 *	0,024 *	0,851 **	0,151 *	0,007 *	0,275 **	0,044 *	0,022 *	0,801 **
Afetivo									
Baixo	5 (62,5)	4 (100,0)	1 (50,0)	3 (37,5)	2 (50,0)	1 (50,0)	5 (62,5)	3 (75,0)	1 (50,0)
Médio	22 (51,2)	27 (87,1)	12 (85,7)	14 (32,6)	20 (64,5)	12 (85,7)	19 (44,2)	24 (77,4)	12 (85,7)
Alto	44 (35,2)	50 (51,0)	17 (58,6)	26 (20,8)	56 (57,1)	20 (69,0)	21 (16,8)	55 (56,1)	23 (79,3)
Valor de p	0,073 **	< 0,001 **	0,158 **	0,175 **	0,676 **	0,332 **	< 0,001 **	0,070 **	0,510 **

Fonte: elaboração própria.

* Teste qui-quadrado de Pearson;

** Teste exato de Fisher.

## Discussão

O estudo foi realizado em cinco centros localizados no Município do Rio de Janeiro, mas quase a metade dos cuidadores morava em outras regiões do estado. Observou-se que parcela importante dos cuidadores de crianças com cegueira e com baixa visão eram jovens (entre 20 e 29 anos) e aquele grupo tinha o menor nível de escolaridade quando comparados aos cuidadores de crianças com baixa visão e sem deficiência visual.

A *Pesquisa Nacional de Saúde* (PNS) estima que cerca de 0,5% da população brasileira de 2 a 9 anos tem algum tipo de deficiência visual ^27^ e seus resultados apontam que, nos domicílios com rendimento de até um salário mínimo, há de 3,9% a 4,4% pessoas residentes com alguma condição associada à baixa visão ou cegueira, o que corresponde a cerca de três vezes mais que os domicílios com rendimento *per capita* de 5 salários mínimos ou mais ^28^. Neste estudo, cerca de 28% das crianças com deficiência visual tinham até 5 anos de idade e aproximadamente 30% dos cuidadores de crianças com deficiência visual reportaram uma renda mensal média inferior a um salário mínimo vigente na época da coleta de campo (BRL 998,00).

Este é um cenário de baixa renda e baixa escolaridade que não emerge como uma situação inédita entre os cuidadores de crianças com alguma deficiência ^15^^,^^16^^,^^28^. Agrava essa realidade o fato de a maioria dos cuidadores de crianças com cegueira (60%) ter deixado de trabalhar após o nascimento, o que pode indicar uma importante dependência de transferências financeiras governamentais. A saída do mercado de trabalho ou a redução do número de horas é recorrente para as famílias de crianças com alguma deficiência. A perda anual de renda por família, nos Estados Unidos, alcança USD 18 mil (cerca de BRL 100 mil) ou em dados agregados para todo o país de USD 14,3 bilhões a 19,2 bilhões (aproximadamente de BRL 78,65 bilhões a BRL 106 bilhões) ^29^. A magnitude desses números reflete a perda de produtividade, que gera custos indiretos que podem afetar sobremaneira a economia do país.

Ao discutirmos tal cenário de resultados, é relevante considerar a intersecção entre saúde, com destaque para expressões de sintomas de atenção à saúde mental, e desigualdades relacionadas ao gênero, renda e escolaridade. Temos mulheres de classes menos privilegiadas e com baixa ou nenhuma escolaridade. Esse retrato - que tem como limite o fato de a pesquisa não ter incluído o componente racial - já revela uma vulnerabilidade social digna de nota. Há que considerar que possíveis chefias femininas de família se dão em função de esgarçamentos das redes de suporte e abandono paterno ^30^.

Outros estudos já registram um retrato de uma chefia de família feminina em função de abandono do pai da criança, nascida ou tornada crônica ou com deficiência, em função de intercorrências/acidentes, gerando pauperização da família e sobrecarga das mulheres no cuidado com a criança ^15^^,^^16^^,^^17^^,^^28^^,^^29^^,^^31^ e a existência de situações de vulnerabilidade à violência de crianças e adolescentes com condições crônicas e/ou deficiências ^32^^,^^33^^,^^34^. Nesse sentido, tal situação não se restringe somente à discussão de mecanismos de proteção financeira, mas também de uma política de apoios que não tangencie e que seja protagonista no acolhimento dos cuidadores de crianças com deficiência visual e suas famílias.

Cabe-nos destacar que a grande dedicação das mulheres ao cuidado de crianças com deficiência não é necessariamente algo relacionado a uma relação direta entre deficiência e questões de saúde mental. Nosso argumento neste artigo visa, primeiramente, gerar um retrato da situação de saúde mental e do apoio social de mulheres-mães de crianças com deficiência visual. Os resultados sugerem que a prevalência de sintomas depressão, ansiedade e estresse entre os cuidadores de crianças com deficiência visual foram superiores à observada entre os cuidadores de crianças sem deficiência.

A avaliação das escalas MOS-SSS e DASS-21 nos trouxe, na perspectiva quantitativa, algumas relações, como menor comprometimento visual, maiores apoios e menores escores de relatos de sintomas de depressão, ansiedade e estresse entre cuidadores de crianças sem deficiência e com baixa visão. Mas, quando se coloca sob foco os cuidadores de crianças com cegueira, as maiores prevalências de depressão, ansiedade e estresse foram independentes dos graus de apoios recebidos. Tais dados são indicativos de compromissos que precisam ser estabelecidos com políticas de cuidado no campo da deficiência, principalmente quando as questões da dependência se apresentam como sustentadoras do cuidado ^35^. Ademais, compreendendo que os resultados acerca dos sintomas de depressão traduzidos pelas escalas representam um corte temporal no qual o cuidador responde ao questionário por meio da relação com seu(sua) entrevistador(a), cabe, portanto, observar que outros fatores relacionados à sua localização social - mulher, de qual raça, classe, território e geração - podem se fazer relevantes quando associados aos seus estados de saúde mental.

Sob esse aspecto, trazemos ainda outras limitações. A inclusão da amostra de cuidadores de crianças com doenças oculares e dos controles do estudo Seiz foi necessária devido à perda de participantes desde o início da pandemia de SARS-CoV-2 em 2020. No entanto, essa amostra seguiu os mesmos critérios de inclusão do Views-QoL Study e os mesmos instrumentos foram aplicados ^23^. A ocorrência de viés de informação (memória) na aplicação dos questionários também deve ser considerada. Em relação à renda, o Critério Brasil foi uma forma de validar a informação do cuidador (dada em faixas salariais) e, assim, minimizar possíveis discrepâncias. Nesse caso, não se observaram diferenças significativas entre ambas. A generalização dos resultados apresentados não é possível devido à realização do estudo somente em centros participantes localizados no Município do Rio de Janeiro. Por isso, pode não representar a realidade brasileira, diversa em termos de renda, acesso e demanda por serviços de saúde e de educação, bem como as características clínicas das crianças. As especificidades da escala DASS-21, como sua subjetividade e a mensuração da frequência de sintomas sem a determinação do diagnóstico de depressão, ansiedade e estresse, requerem que os resultados sejam interpretados com cautela. Apesar de tais limitações, este estudo indica a importância da aplicação da escala como rastreio para identificar a presença de tais sintomas entre cuidadores de crianças com deficiência visual.

Destacamos que, conforme definimos anteriormente, a deficiência como característica pode ser desqualificada e levar a um descrédito da criança por barreiras atitudinais relacionadas aos estigmas e preconceitos. Nessa linha de argumentação, faz sentido que possamos discutir a depressão de mulheres, mães de crianças com deficiência, compreendendo que o cuidado de que falamos se refere a uma ética do cuidado como política, e abrir sua relação com o apoio social. A restrição de relações sociais opera como fator de risco à saúde, tão perniciosa como o fumo, a pressão arterial elevada, a obesidade e a ausência de atividade física ^21^. Podemos inferir que as cargas de cuidado da mulher-mãe de uma criança com deficiência podem comprometer - caso ela esteja com suas redes de apoio muito restritas - um movimento de trocas pessoais e afetivas, podendo levar a um isolamento de redes familiares, de amigos e vizinhança. Esse processo de isolamento tem, no trabalho de Sluzki ^20^, um outro nome: desvitalização do intercâmbio interpessoal. Desvitalizar significa reduzir a vitalidade e intercâmbio, a troca e a reciprocidade. Segundo o autor, essa desvitalização contribui para um “círculo vicioso desintegrador das redes sociais”.

A falta de apoio em diferentes contextos, como o comunitário e na própria família do cuidador, é relatada, em alguns estudos, como uma dificuldade das mães de crianças com deficiência múltipla ^36^^,^^37^. Inclusive, essas cuidadoras mencionam a indisponibilidade para participar de atividades sociais diante de uma rotina de cuidados que lhes toma tempo, contrariamente ao que ocorre com aquelas mães que recebem apoio de maridos, familiares e vizinhos e que têm espaço nas suas rotinas para participar com mais facilidade dessas atividades. Em síntese, reiteramos que a presença de laços de ajuda mútua, com alcance de apoio social, pode operar como fator protetor e a sua ausência ou esgarçamento/enfraquecimento podem incrementar o risco de adoecimentos de outras ordens, interligados ao declínio da rede ^20^.

## Conclusão

Este trabalho é parte de uma pesquisa que enfrentou os desafios da pandemia de SARS-CoV-2, o que exigiu do grupo de pesquisadores a criatividade de trazer, para a construção metodológica, uma alternativa que permitisse a viabilidade do estudo. Logo, o que pode ser encarado como limite também traz a possibilidade de reconhecer a pesquisa e seus métodos como vivos, no desafio de enfrentar cenários da vida real e imprevistos, sem significar pouco rigor científico.

Portanto, não é a deficiência que produz barreiras ao cuidado, nem atribui graus de ansiedade, depressão e estresse, mas ela, em suas múltiplas expressões, denuncia as desigualdades na ausência ou precariedade de suporte, como algo legítimo da existência humana, para quem cuida ou recebe cuidado. Precisamos ter clareza que os casos de deficiência visual se cronificam não pela deficiência exclusivamente, ou por causalidade, mas pelas associações mais finas, “responsáveis” pelas mães apresentarem situações de sintomas de saúde mental dignas de atenção. Ou seja, a combinação entre ter um filho com deficiência, associada a um afrouxamento dos laços de apoio social, e um Estado que não ofereça políticas de cuidado garantindo a interdependência positiva de suportes é que precisa ser enfrentada no campo da luta por políticas de inclusão e suporte social, de base intersetorial. Faz-se urgente esse letramento social e político da deficiência para que interpretemos os dados, os limites e as pessoas que existem por trás dos números.
